# *In vitro* antimicrobial activity of nitroxoline against uropathogens isolated from China

**DOI:** 10.1093/jacamr/dlaf012

**Published:** 2025-02-04

**Authors:** Xiaofei Yi, Xin Chen, Yanyan Lu, Jianfeng Zhang, Jinhong Chen, Minggui Wang, Xiaogang Xu

**Affiliations:** Institute of Antibiotics, Huashan Hospital, Fudan University, Shanghai 20040, China; Institute of Antibiotics, Huashan Hospital, Fudan University, Shanghai 20040, China; Institute of Antibiotics, Huashan Hospital, Fudan University, Shanghai 20040, China; Institute of Antibiotics, Huashan Hospital, Fudan University, Shanghai 20040, China; Institute of Antibiotics, Huashan Hospital, Fudan University, Shanghai 20040, China; Institute of Antibiotics, Huashan Hospital, Fudan University, Shanghai 20040, China; Institute of Antibiotics, Huashan Hospital, Fudan University, Shanghai 20040, China

## Abstract

**Background and objectives:**

The antimicrobial nitroxoline is used in treating uncomplicated urinary tract infections (UTIs) in some European countries. *In vitro* antimicrobial data on uropathogens from China are lacking. To investigate the activity of nitroxoline *in vitro* against uropathogens isolated from Chinese patients.

**Methods:**

MICs of nitroxoline were determined using broth microdilution of 229 bacterial isolates of 10 species including *Acinetobacter baumannii* derived from urinary sample. The EUCAST susceptibility breakpoint for *Escherichia coli* (16 mg/L) was applied for all isolates. The MBC for *A. baumannii* (*n* = 34) was determined, with the MBC defined as the nitroxoline concentration at which a 99.9% reduction in the initial inoculum was observed. Time-kill curves of the two isolates of *A. baumannii* were tested over a 24 h period.

**Results:**

Except for *Pseudomonas aeruginosa*, most isolates were susceptible to nitroxoline. The MIC50/90 values of nitroxoline for *E. coli*, *Klebsiella pneumoniae* and *Proteus mirabilis* were 4/8, 8/32 and 8/16 mg/L, respectively. Among the Gram-negative isolates, nitroxoline demonstrated a better inhibitory effect against *A. baumannii* with an MIC50/90 value of 2/2 mg/L. The MBC of *A. baumannii* was equal to the MIC or one dilution higher. The time-kill curves of *A. baumannii* displayed concentration-dependent killing.

**Conclusions:**

Nitroxoline showed excellent *in vitro* activity against uropathogens isolated from China and may be a good option for uncomplicated UTIs caused by *A. baumannii*, which are more challenging and have few clinical options. Further *in vivo* efficacy studies are needed.

## Introduction

Urinary tract infections (UTIs) are common among humans but disproportionately affect women, with ∼50% of women experiencing a UTI throughout their lifetime.^1^ Although UTIs are prevalent among young sexually active populations, the risk of UTIs in women generally increases with age; thus, the risk of UTIs in post-menopausal and older women is significantly increased.^1–3^ UTIs can be caused by various bacteria and fungi; the most frequent pathogens include *Escherichia coli* and other species of Gram-negative bacteria (such as *Klebsiella pneumoniae*, *Proteus mirabilis*, *Enterobacter cloacae*, *Pseudomonas aeruginosa* and *Acinetobacter baumannii*) and Gram-positive bacteria (such as *Enterococcus faecium*, *E. faecalis* and *Staphylococcus aureus*).^1,4^

Increased resistance of uropathogens to orally administered antimicrobials has complicated the management of UTI in outpatients.^5^ Particularly, carbapenem-resistant Gram-negative bacilli (CRGNB) infections are increasing over time, for which antimicrobial treatment options are limited.^6^ Such difficult-to-treat CRGNB infections result in a heavy healthcare burden and high mortality rate.^6,7^ In this context, nitroxoline (5-nitro-8-hydroxyquinoline), an oral antimicrobial agent first described in the 1950s,^8^ has received renewed attention in managing uncomplicated UTIs in Germany. Nitroxoline is a quinoline derivative that does not belong to any known group of antimicrobials owing to its unique mechanism of action, which depends almost exclusively on its chelating properties.^9^ It is based on the chelation and sequestration of biologically important divalent ions that subsequently result in bacterial death. The chelating property of nitroxoline can inhibit the function of bacterial RNA polymerase and inhibit the biofilm formation of a variety of pathogenic microorganisms, thereby reducing the adhesion of bacteria to the bladder.^10–12^ In 2016, the EUCAST proposed the clinical breakpoint of nitroxoline (susceptible ≤16 mg/L), which was limited to *E. coli* and uncomplicated UTIs,^13^ since oral administration of the standard dose of 250 mg every 8 h results in higher urinary concentrations and lower systemic concentrations.^14^ Nitroxoline is active *in vitro* against both Gram-positive and Gram-negative bacteria as well as fungal pathogens.^15^ Among Gram-negative organisms, nitroxoline exhibits good activity against *Enterobacterales* and *Acinetobacter* but not *Pseudomonas* spp.^15–17^ Furthermore, currently, nitroxoline is used in treating acute and recurrent UTIs in adults and children only in certain European countries and is not used in China. However, uropathogens in China differ in terms of species and antimicrobial resistance (AMR).^18,19^ The top five pathogens for uropathogens in Europe are *E. coli*, *K. pneumoniae*, *P. mirabilis*, *E. faecalis* and *S. saprophyticus*,^19^ while in China, the main uropathogens include *E. coli*, *K. pneumoniae*, *E. faecalis*, and some non-fermenting bacteria (such as *P. aeruginosa*).^18^ In terms of AMR, the issue of AMR in China is relatively severe, with an increasing number of strains resistant to fluoroquinolones and cephalosporins. Especially *A. baumannii*, which ranks ninth among uropathogens in China with an isolation rate of 1.7%, has shown an increasing trend of resistance to most antimicrobial drugs over the years. According to statistical data from 2021, the resistance rate of *A. baumannii* to antimicrobial drugs such as carbapenems, third-generation cephalosporins and ciprofloxacin were all ≥41.8%.^18^ At present, *in vitro* antimicrobial data for uropathogens from China are lacking.

Thus, in the present study, we aimed to investigate the activity of nitroxoline *in vitro* against uropathogens in China and predict its application prospects in Chinese patients with UTIs. *A. baumannii* was used as they represent pathogen to study the bactericidal effect of nitroxoline.

## Materials and methods

### Ethics

The bacterial strains used in this study were obtained from the strain bank of the Institute of Antibiotics of Huashan Hospital. Our study received approval from the Ethics Committee of Huashan Hospital (approval no. KY2017-274).

### Bacterial strains

The collection consisted of 229 clinical bacterial isolates derived from urinary samples of patients diagnosed with a UTI at Huashan Hospital in 2022. The isolates included *E. coli* (*n* = 30, 10 strains were carbapenem resistant, and 20 were carbapenem susceptible), *K. pneumoniae* (*n* = 30, 10 strains were carbapenem resistant, and 20 were carbapenem susceptible), *P. mirabilis* (*n* = 20), *E. cloacae* (*n* = 20), *A. baumannii* (*n* = 34, 17 strains were carbapenem resistant, and 17 were carbapenem susceptible), *P. aeruginosa* (*n* = 42, 12 strains were carbapenem resistant, and 32 were carbapenem susceptible), *E. faecium* (*n* = 10), *E. faecalis* (*n* = 20), *Staphylococcus epidermidis* (*n* = 10) and *S. aureus* (*n* = 13). *E. coli* ATCC 25922 (the only strain with a nitroxoline quality control range defined by EUCAST) was used as a quality control. The selection of carbapenem-resistant and susceptible strains was based on the definition of carbapenem resistance, which refers to the resistance to one or more carbapenem antibiotics.^20,21^

### Determination of the MIC

The MICs of meropenem, imipenem and levofloxacin were determined using the VITEK 2 system (bioMérieux, Craponne, France). Nitroxoline MICs were determined using broth microdilution, following the CLSI recommendations. Nitroxoline powder was dissolved in DMSO (Sangon Biotech, Shanghai, China), and dilutions were prepared in sterile water. The concentration of DMSO was adjusted to be consistent across antibiotic concentrations. The isolates were tested in 96 well plates with final nitroxoline concentrations ranging from 0.5 to 256 mg/L. The EUCAST susceptibility breakpoint for *E. coli* (16 mg/L) was applied for all isolates in this study.

### Determination of the MBC

The MBCs of nitroxoline were determined for *A. baumannii* (*n* = 34), *E. coli* (*n* = 5), *K. pneumoniae* (*n* = 5) and *E. coli* ATCC25922 by plating the well contents from the MIC test on LB agar. A volume of 100 μL liquid was removed from the wells in the microtiter plates where no growth was observed after a 24 h incubation at 37°C, washed two times with 0.85% sodium chloride (NaCl) solution and spread on the surface of LB agar plates. The colonies were counted after 24 h of incubation at 37°C. The initial number of colonies was recorded by plating the plates after dilution with the inoculum solution. Because the limit of detection for this technique is 10 cfu/mL, the absence of any growth on an LB agar plate indicated that the concentration was below this value. The MBC was deemed the minimum concentration of an antimicrobial agent capable of inactivating >99.9% of the bacteria present. Three replicates were analysed for each strain and antimicrobial compound. The surviving fraction of cells was determined using the ratio of cfu before nitroxoline treatment. Bactericidal activity was defined by a reduction of ≥3 log 10 cfu/mL compared with the initial inoculum. The regrown colonies were retested for MICs to compare them with the original strain.

### Time-kill curve

The bactericidal activity of nitroxoline against the two strains of *A. baumannii* was determined in triplicates. Three to five single colonies were selected from LB agar plates and adjusted to 0.5 McFarland. The bacterial solution was diluted in 25 mL LB medium at a ratio of 1:100 to achieve a concentration of ∼10^6^ cfu/mL. Nitroxoline was added at concentrations ranging from 1 to 8 mg/L (0.5–4  ×  MIC). After 0, 1, 2, 4, 8, 12 and 24 h of incubation at 37°C, aliquots (1 mL) were withdrawn, and the cells were washed and serially diluted in 0.85% NaCl. Subsequently aliquots (100 μL) were withdrawn and spread on LB agar plates for the viable count determination. Colonies were counted after incubation for 24 h at 37°C.

## Results

### *In vitro* antibacterial effect

The MICs of nitroxoline, meropenem, imipenem and levofloxacin for all isolates are summarized in Table 1, and the MIC values are listed in Table S1 (available as Supplementary data at *JAC-AMR* Online). Except for *P. aeruginosa* and *K. pneumonia*, all nitroxoline MIC90 values were ≤16 mg/L (susceptibility breakpoint of 16 mg/L). When comparing all tested species, nitroxoline showed a better inhibitory effect against *A. baumannii* (sensitivity of 100%, MIC50/90 of 2/2 mg/L and MIC range of 1–4 mg/L) among the Gram-negative isolates. All *E. coli*, *P. mirabilis* and four species of Gram-positive bacterial isolates were 100% susceptible to nitroxoline, and the MIC50/90 values of nitroxoline were 4/8, 8/16, 1/2, 2/4, 8/8 and 8/16 mg/L, respectively. The sensitivity rates of nitroxoline for *K. pneumoniae*, *E. cloacae* and *P. aeruginosa* were 83.3%, 95% and 23.81%, respectively, and those MIC50/90 for nitroxoline were 8/32, 8/16 and 36/64 mg/L, respectively. Based on the MIC values, there was nodifference in activity between the carbapenem-resistant and carbapenem-susceptible isolates of *E. coli*, *K. pneumoniae*, *P. aeruginosa* and *A. baumannii*. The MIC50/90 values for nitroxoline against both carbapenem-resistant and carbapenem-susceptible strains were 8/32. *E. coli*, *K. pneumoniae*, *P. mirabilis*, carbapenem-resistant *A. baumannii*, *E. faecalis* and *S. epidermidis* showed high MIC50/90 values for levofloxacin and resistance rates >70%. The MIC of nitroxoline against the quality control strain *E. coli* ATCC 25922 was 2 mg/L, which was within the accepted range defined by the EUCAST.

**Table 1.** MICs of nitroxoline, meropenem, imipenem and levofloxacin against uropathogens^a^

			Nitroxoline					
			MIC50/90 (mg/L)	MIC range (mg/L)	Susceptibility (%)	Meropenem MIC50/90 (mg/L)	Imipenem MIC50/90 (mg/L)	Levofloxacin MIC50/90 (mg/L)
	Comment							
*E. coli*	*n* = 30		4/8	2–16	(30/30) 100.00	≤0.06/16	16/>16	>8/>8
	CR	*n* = 10	8/8	2–16	(10/10) 100.00	16/>16	16/>16	>8/>8
	CS	*n* = 20	4/8	2–8	(20/20) 100.00	≤0.06/≤0.06	≤0.25/≤0.25	>8/>8
*K. pneumoniae*	*n* = 30		8/32	1–64	(25/30) 83.30	0.12/>16	≤0.25/>16	>8/>8
	CR	*n* = 10	4/8	2–16	(10/10) 100.00	>16/>16	>16/>16	>8/>8
	CS	*n* = 20	16/32	1–64	(15/20) 75.00	≤0.06/0.5	≤0.25/≤0.25	>8/>8
*A. baumannii*	*n* = 34		2/2	1–4	(34/34) 100.00	2/>16	≤0.25/>16	≤0.12/>8
	CR	*n* = 17	2/2	1–4	(17/17) 100.00	>16/>16	>16/>16	>8/>8
	CS	*n* = 17	2/2	1–4	(17/17) 100	1/2	≤0.25/≤0.25	≤0.12/0.5
*P. aeruginosa*	*n* = 42		32/64	16–64	(10/42) 23.81	0.5/>8	2/>16	0.5/>8
	CR	*n* = 12	32/64	16–64	(3/12) 25	8/>8	>16/>16	4/>8
	CS	*n* = 30	32/32	16–64	(7/30) 23.33	≤0.25/1	2/2	0.5/1
*P. mirabilis*	*n* = 20		8/16	2–16	(20/20) 100.00	≤0.25/≤0.25	1/2	>8/>8
*E. cloacae*	*n* = 20		8/16	8–32	(19/20) 95.00	≤0.25/1	0.5/2	1/>8
*S. epidermidis*	*n* = 10		1/2	≤0.5–1	(10/10) 100	NA	NA	4/>4
*S. aureus*	*n* = 13		2/4	2–4	(13/13) 100	NA	NA	0.25/>4
*E. faecalis*	*n* = 10		8/8	4–16	(10/10) 100	NA	NA	>4/>4
*E. faecium*	*n* = 20		8/16	4–16	(20/20) 100	NA	NA	1/>4

^a^CR, carbapenem resistant; CS, carbapenem susceptible; NA, not applicable.

### *In vitro* bactericidal effect

**Figure 1.**
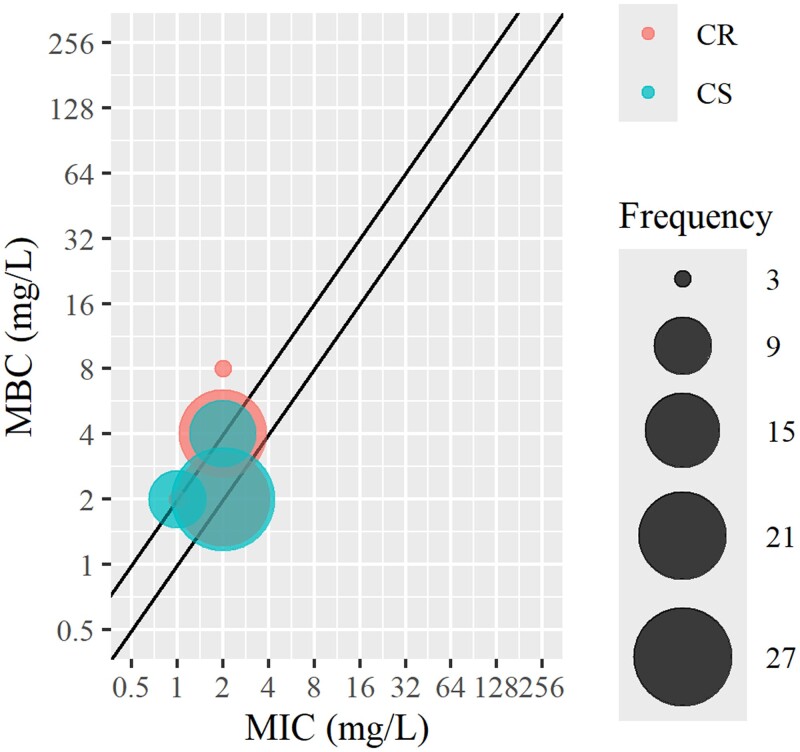
MICs and MBCs of nitroxoline for 34 strains of *A. baumannii*. The lines represent *y* = 1*x* and *y* = 2*x*. The coordinates of the balloons represent the MIC and MBC values from one repetition, and the size of the balloons correlate to the counts of their occurrence in all repetitions. Three replicates were performed for each strain. CR, carbapenem-resistant isolates; CS, carbapenem-susceptible isolates.

The MBC values of nitroxoline for the 34 *A. baumannii* isolates were 2 mg/L (65%, 22/34) and 4 mg/L (45%, 11/34) in 97% of the isolates. The MBC was high (8 mg/L) in only one strain. The distribution and comparison with MIC values are shown in Figure 1. The values for the MBCs were equal to or one dilution higher than those for the MICs. There was no correlation between the nitroxoline MBCs and carbapenem MICs, and low nitroxoline MBCs were recorded for isolates that were both carbapenem resistant and carbapenem susceptible. The quality control strain, *E. coli* ATCC 25922, showed a nitroxoline MBC of 64 mg/L, which was five times higher than that of the MIC.

**Figure 2.**
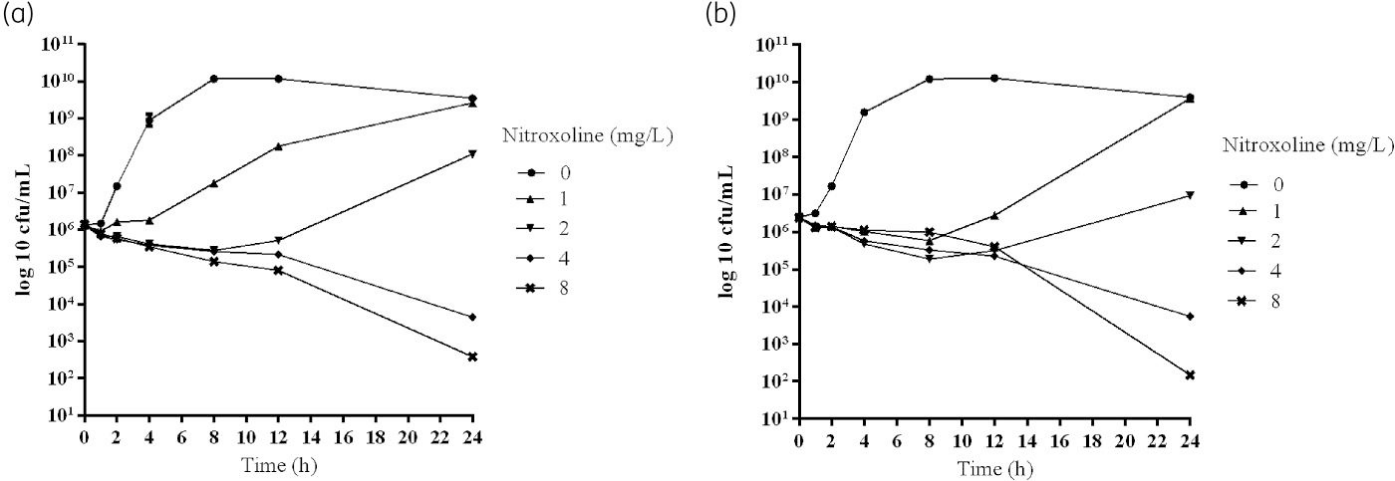
*In vitro* kill curves of nitroxoline at 0, 1, 2, 4 and 8 mg/L of two strains of *A. baumanni*: (a) aba4 and (b) aba8.

To further confirm the *in vitro* bactericidal effect of nitroxoline, its killing kinetics was studied in two strains of *A. baumannii* (aba4 and aba8, both of which had an MIC of 2 mg/L). As shown in Figure 2, concentration-dependent killing and significant reductions in viable bacterial counts were observed after 24 h of exposure of the bacteria to nitroxoline at concentrations above the MIC. The maximum reduction in viable counts was 3 log10 cfu/mL for 8 mg/L nitroxoline when compared with the initial inocula, and the antibacterial effect of nitroxoline on the two strains was determined to be bacteriostatic at the test concentrations.

## Discussion

With increasing AMR, especially in Gram-negative bacteria, UTI treatment has become increasingly difficult. Therefore, older and long-unused antimicrobials, including nitroazoline, nitrofurantoin and mecillinam, have received renewed attention recently because of their high activity against many resistant pathogens and their ability to achieve high urinary concentrations after oral administration.^12,15–17,22^ However, systemic concentrations are comparatively low, resulting in nitroxoline being only approved for uncomplicated UTIs.^14^

In this study, the high resistance rate to levofloxacin indicates that it is unsuitable for treating UTIs in China. Nitroxoline showed excellent *in vitro* activity against most isolates. If the EUCAST susceptibility breakpoint for *E. coli* (16 mg/L) was applied, 100% of the *A. baumannii* (34/34), *E. coli* (30/30), *P. mirabilis* (20/20) and four species of Gram-positive bacteria (53/53) isolates would be classified as susceptible to nitroxoline. The sensitivity rates to nitroxoline of *K. pneumoniae* and *E. cloacae* were 83.3% (25/30) and 95% (29/20), respectively. *P. aeruginosa* showed tolerance to nitroxoline with a sensitivity rate of only 23.1% (10/42). In a previous study from Germany,^15,16,22^ 100% of *A. baumannii* (34/34) and *E. coli* isolates (30/30) were susceptible to nitroxoline, and the sensitivity rates for *K. pneumoniae*, *E. cloacae* and *P. aeruginosa* were 95.7% (44/46), 76.9% (3/13) and 24.3% (9/37), respectively, similar to the results of our study. Currently, there is a lack of research on the mechanisms of resistance of *P. aeruginosa* to nitroxoline. For high MIC *K. pneumoniae*, a previous study^23^ has suggested that this may be due to the ability of these bacteria to produce a capsule layer that protects them from toxic substances.^24^ Additionally, *K. pneumoniae* reduces the absorption and avoids contact with antimicrobial drugs through various mechanisms, such as decreasing outer membrane permeability or increasing efflux pumps production. Based on the MIC of nitroxoline, meropenem and imipenem in *E. coli*, *K. pneumoniae*, *P. aeruginosa* and *A. baumannii*, there was no correlation between nitroxoline MICs and carbapenem MICs (Table 1), and low nitroxoline MICs were recorded for isolates with both carbapenem resistance and carbapenem sensitivity, similar to the study of Fuchs and Hamprecht.^22^ The tested Gram-positive species also showed MICs well below the breakpoint established so far only for *E. coli.* Therefore, nitroxoline has a broad antimicrobial spectrum and could be a promising oral treatment option for uncomplicated UTIs.

Among the Gram-negative bacteria we tested, by comparing MIC values, we found that nitroxoline had better *in vitro* activity against *A. baumannii*. The treatment of UTIs caused by multidrug-resistant *A. baumannii* is even more difficult because no other oral antimicrobial option exists. Most isolates are resistant to trimethoprim/sulfamethoxazole and fluoroquinolones, and there are fewer clinical drug options against carbapenem-resistant *A. baumannii.*^16^ Therefore, we further studied the bactericidal effect of nitroxoline on 34 strains of *A. baumannii in vitro*. Low MBC values of 34 strains of *A. baumannii* were obtained, and the MBC values were equal to or one dilution higher than the MIC values, which is consistent with the primary bacteriostatic nature.^15^ Sobke *et al.*^15^ determined the MBC (32 mg/L) of ATCC 25922, which was at least four times higher than the MIC, similar to the MBC (64 mg/L) in our study. However, the MBCs of *A. baumannii* have not yet been reported. The time-kill curve for *A. baumannii* further confirmed the *in vitro* bactericidal activity of nitroxoline.

Our study had some limitations. First, the sample size was small, which may have limited the representativeness of our results. Second, *in vitro* activity does not necessarily correlate with clinical or microbiological success *in vivo*, and the *in vivo* antimicrobial effects of nitroxoline require further study.

### Conclusion

Nitroxoline showed a broad antimicrobial spectrum, and its activity against *A. baumannii* was promising, not only when applying the current EUCAST breakpoint for *E. coli* (MIC90 < 16 mg/L) but also when considering the pharmacokinetics of nitroxoline. High urinary concentrations were observed after oral treatment with nitroxoline (5.4 mg/L of the unconjugated form and 210.6 mg/L of the conjugated form^25^). Nitroxoline has strong bactericidal activity against *A. baumannii*, with MBCs only one or two log2 dilutions higher than their MICs. These results indicate that nitroxoline showed excellent *in vitro* activity against most uropathogens isolated from China, except for *P. aeruginosa*. It shows potential as a treatment option for uncomplicated UTIs caused by *A. baumannii*, a pathogen that presents significant therapeutic challenges and has limited treatment options. Additional *in vivo* studies are needed to confirm these findings.

## Supplementary Material

dlaf012_Supplementary_Data

## Supplementary data

Table S1 is available as Supplementary data at *JAC-AMR* Online.
