# Supplementary material for: In vitro antimicrobial activity of nitroxoline against uropathogens isolated from China
Source: JAC Antimicrob Resist. 2025 Feb 4;7(1):dlaf012. doi: 10.1093/jacamr/dlaf012 (PMC11791683; doi:10.1093/jacamr/dlaf012)
Supplement: dlaf012_Supplementary_Data [file dlaf012_supplementary_data.docx]

**Supplementary data**

Table S1. MICs of nitroxoline, meropenem, imipenem and levofloxacin against 229 uropathogens.^b^

| **Species** | **Number** | **Comment** | **Gram** | **Nitroxoline**  **MIC (mg/L)** | **Meropenem**  **MIC (mg/L)** | **Imipenem**  **MIC (mg/L)** | **Levofloxacin**  **MIC (mg/L)** |
| --- | --- | --- | --- | --- | --- | --- | --- |
| *E. coli (n=30)* | eco1 | CR | GN | 8 | >8 | >8 | >8 |
|  | eco2 | CR | GN | 16 | 8 | >8 | >8 |
|  | eco3 | CR | GN | 16 | >16 | >16 | >8 |
|  | eco4 | CR | GN | 8 | >8 | >8 | >8 |
|  | eco5 | CR | GN | 8 | 16 | 16 | >8 |
|  | eco6 | CR | GN | 8 | 16 | 16 | >8 |
|  | eco7 | CR | GN | 4 | 16 | 8 | >8 |
|  | eco8 | CR | GN | 8 | 16 | 8 | >8 |
|  | eco9 | CR | GN | 8 | 16 | 16 | >8 |
|  | eco10 | CR | GN | 2 | 16 | >16 | >8 |
|  | eco11 | CS | GN | 8 | ≤0.06 | ≤0.25 | ≤0.12 |
|  | eco12 | CS | GN | 4 | ≤0.06 | ≤0.25 | >8 |
|  | eco13 | CS | GN | 2 | ≤0.06 | ≤0.25 | 1 |
|  | eco14 | CS | GN | 4 | ≤0.06 | ≤0.25 | >8 |
|  | eco15 | CS | GN | 4 | ≤0.06 | ≤0.25 | >8 |
|  | eco16 | CS | GN | 4 | ≤0.06 | ≤0.25 | >8 |
|  | eco17 | CS | GN | 8 | ≤0.06 | ≤0.25 | >8 |
|  | eco18 | CS | GN | 4 | ≤0.06 | ≤0.25 | >8 |
|  | eco19 | CS | GN | 2 | ≤0.06 | ≤0.25 | 1 |
|  | eco20 | CS | GN | 8 | ≤0.06 | ≤0.25 | >8 |
|  | eco21 | CS | GN | 8 | ≤0.06 | ≤0.25 | 8 |
|  | eco22 | CS | GN | 2 | ≤0.06 | ≤0.25 | 8 |
|  | eco23 | CS | GN | 4 | ≤0.06 | ≤0.25 | >8 |
|  | eco24 | CS | GN | 4 | ≤0.06 | ≤0.25 | >8 |
|  | eco25 | CS | GN | 4 | ≤0.06 | ≤0.25 | ≤0.12 |
|  | eco26 | CS | GN | 4 | ≤0.06 | ≤0.25 | >8 |
|  | eco27 | CS | GN | 8 | ≤0.06 | ≤0.25 | 1 |
|  | eco28 | CS | GN | 8 | ≤0.06 | ≤0.25 | >8 |
|  | eco29 | CS | GN | 4 | ≤0.06 | ≤0.25 | >8 |
|  | eco30 | CS | GN | 8 | ≤0.06 | ≤0.25 | >8 |
| *K. pneumoniae (n=30)* | kpn1 | CR | GN | 4 | >16 | >16 | >8 |
|  | kpn2 | CR | GN | 8 | >16 | >16 | >8 |
|  | kpn3 | CR | GN | 8 | >16 | >16 | >8 |
|  | kpn4 | CR | GN | 2 | >16 | >16 | 4 |
|  | kpn5 | CR | GN | 4 | >16 | >16 | >8 |
|  | kpn6 | CR | GN | 16 | >16 | >16 | >8 |
|  | kpn7 | CR | GN | 2 | >16 | >16 | >8 |
|  | kpn8 | CR | GN | 4 | >16 | >16 | >8 |
|  | kpn9 | CR | GN | 4 | >16 | >16 | >8 |
|  | kpn10 | CR | GN | 8 | >16 | >16 | 4 |
|  | kpn11 | CS | GN | 8 | 2 | ≤0.25 | 4 |
|  | kpn12 | CS | GN | 4 | 0.5 | ≤0.25 | >8 |
|  | kpn13 | CS | GN | 64 | ≤0.06 | ≤0.25 | 8 |
|  | kpn14 | CS | GN | 16 | ≤0.06 | ≤0.25 | >8 |
|  | kpn15 | CS | GN | 16 | ≤0.06 | ≤0.25 | >8 |
|  | kpn16 | CS | GN | 1 | 0.5 | ≤0.25 | >8 |
|  | kpn17 | CS | GN | 16 | 0.12 | ≤0.25 | 4 |
|  | kpn18 | CS | GN | 16 | 0.12 | ≤0.25 | >8 |
|  | kpn19 | CS | GN | 32 | 0.12 | ≤0.25 | >8 |
|  | kpn20 | CS | GN | 16 | ≤0.06 | ≤0.25 | >8 |
|  | kpn21 | CS | GN | 2 | ≤0.06 | ≤0.25 | >8 |
|  | kpn22 | CS | GN | 32 | 0.12 | ≤0.25 | >8 |
|  | kpn23 | CS | GN | 16 | ≤0.06 | ≤0.25 | >8 |
|  | kpn24 | CS | GN | 16 | 0.12 | ≤0.25 | >8 |
|  | kpn25 | CS | GN | 32 | ≤0.06 | ≤0.25 | >8 |
|  | kpn26 | CS | GN | 16 | ≤0.06 | ≤0.25 | 8 |
|  | kpn27 | CS | GN | 8 | ≤0.06 | ≤0.25 | >8 |
|  | kpn28 | CS | GN | 64 | ≤0.06 | ≤0.25 | >8 |
|  | kpn29 | CS | GN | 4 | 1 | ≤0.25 | >8 |
|  | kpn30 | CS | GN | 16 | 0.12 | ≤0.25 | 8 |
| *A. baumannii (n=34)* | aba1 | CR | GN | 2 | >8 | >8 | >8 |
|  | aba2 | CR | GN | 2 | >8 | >8 | >8 |
|  | aba3 | CS | GN | 1 | 0.5 | ≤0.25 | 0.5 |
|  | aba4 | CS | GN | 1 | ≤0.25 | ≤0.25 | ≤0.12 |
|  | aba5 | CR | GN | 2 | >8 | >8 | >8 |
|  | aba6 | CS | GN | 1 | ≤0.25 | ≤0.25 | ≤0.12 |
|  | aba7 | CR | GN | 2 | >8 | >8 | >8 |
|  | aba8 | CR | GN | 1 | >8 | >8 | >8 |
|  | aba9 | CS | GN | 1 | 0.5 | ≤0.25 | 0.5 |
|  | aba10 | CS | GN | 2 | ≤0.25 | ≤0.25 | ≤0.12 |
|  | aba11 | CS | GN | 2 | 0.5 | ≤0.25 | ≤0.12 |
|  | aba12 | CS | GN | 2 | ≤0.25 | ≤0.25 | ≤0.12 |
|  | aba13 | CR | GN | 2 | >8 | >8 | >8 |
|  | aba14 | CR | GN | 2 | >8 | >8 | ≤0.12 |
|  | aba15 | CR | GN | 2 | >8 | >8 | ≤0.12 |
|  | aba16 | CS | GN | 2 | ≤0.25 | ≤0.25 | ≤0.12 |
|  | aba17 | CS | GN | 2 | ≤0.25 | ≤0.25 | ≤0.12 |
|  | aba18 | CS | GN | 2 | ≤0.25 | ≤0.25 | ≤0.12 |
|  | aba19 | CR | GN | 2 | >8 | >8 | >8 |
|  | aba20 | CR | GN | 2 | >8 | >8 | >8 |
|  | aba21 | CS | GN | 2 | ≤0.25 | ≤0.25 | ≤0.12 |
|  | aba22 | CS | GN | 2 | ≤0.25 | ≤0.25 | ≤0.12 |
|  | aba23 | CR | GN | 2 | >8 | >8 | >8 |
|  | aba24 | CR | GN | 4 | >8 | >8 | >8 |
|  | aba25 | CS | GN | 2 | ≤0.25 | ≤0.25 | ≤0.12 |
|  | aba26 | CS | GN | 2 | ≤0.25 | ≤0.25 | ≤0.12 |
|  | aba27 | CR | GN | 2 | >8 | >8 | >8 |
|  | aba28 | CR | GN | 4 | >8 | >8 | >8 |
|  | aba29 | CR | GN | 2 | >8 | >8 | ≤0.12 |
|  | aba30 | CS | GN | 1 | ≤0.25 | ≤0.25 | ≤0.12 |
|  | aba31 | CR | GN | 2 | >8 | >8 | 4 |
|  | aba32 | CS | GN | 1 | ≤0.25 | ≤0.25 | ≤0.12 |
|  | aba33 | CS | GN | 2 | ≤0.25 | ≤0.25 | ≤0.12 |
|  | aba34 | CR | GN | 2 | >8 | >8 | >8 |
| *P. aeruginosa (n=42)* | pae1 | CR | GN | 32 | 8 | >8 | >8 |
|  | pae2 | CS | GN | 32 | 1 | 1 | 1 |
|  | pae3 | CS | GN | 32 | 0.5 | 1 | 1 |
|  | pae4 | CR | GN | 64 | >8 | >8 | 4 |
|  | pae5 | CS | GN | 32 | ≤0.25 | 2 | 0.5 |
|  | pae6 | CS | GN | 32 | 0.5 | 2 | 2 |
|  | pae7 | CS | GN | 64 | 1 | ≤0.25 | 4 |
|  | pae8 | CR | GN | 16 | 4 | >8 | 4 |
|  | pae9 | CR | GN | 64 | >8 | >8 | 4 |
|  | pae10 | CS | GN | 32 | ≤0.25 | 2 | 1 |
|  | pae11 | CS | GN | 32 | 2 | 2 | 0.5 |
|  | pae12 | CS | GN | 32 | ≤0.25 | 2 | 0.5 |
|  | pae13 | CS | GN | 16 | ≤0.25 | 2 | ≤0.12 |
|  | pae14 | CR | GN | 64 | 4 | >8 | 1 |
|  | pae15 | CR | GN | 32 | >8 | >8 | >8 |
|  | pae16 | CS | GN | 32 | 1 | 2 | 0.25 |
|  | pae17 | CS | GN | 32 | ≤0.25 | 2 | 2 |
|  | pae18 | CS | GN | 32 | 2 | 2 | 0.5 |
|  | pae19 | CS | GN | 32 | ≤0.25 | 2 | 2 |
|  | pae20 | CS | GN | 32 | ≤0.25 | ≤0.25 | ≤0.12 |
|  | pae21 | CS | GN | 32 | ≤0.25 | 2 | 1 |
|  | pae22 | CS | GN | 16 | ≤0.25 | 2 | 0.25 |
|  | pae23 | CR | GN | 32 | 2 | >8 | 0.5 |
|  | pae24 | CS | GN | 16 | ≤0.25 | 2 | 1 |
|  | pae25 | CR | GN | 64 | 0.5 | >8 | 0.5 |
|  | pae26 | CS | GN | 16 | ≤0.25 | 2 | 0.25 |
|  | pae27 | CR | GN | 32 | 0.5 | 8 | 0.5 |
|  | pae28 | CS | GN | 16 | 1 | 2 | 0.25 |
|  | pae29 | CR | GN | 16 | >8 | >8 | >8 |
|  | pae30 | CS | GN | 32 | ≤0.25 | 2 | 0.5 |
|  | pae31 | CR | GN | 16 | >8 | >8 | >8 |
|  | pae32 | CS | GN | 16 | ≤0.25 | 2 | 0.25 |
|  | pae33 | CS | GN | 32 | 2 | 2 | 0.25 |
|  | pae34 | CS | GN | 32 | 0.5 | 2 | 0.5 |
|  | pae35 | CS | GN | 16 | ≤0.25 | 1 | 0.5 |
|  | pae36 | CS | GN | 32 | ≤0.25 | 2 | 0.5 |
|  | pae37 | CS | GN | 32 | ≤0.25 | 2 | 1 |
|  | pae38 | CS | GN | 32 | 1 | 1 | 0.25 |
|  | pae39 | CS | GN | 32 | 1 | 2 | 1 |
|  | pae40 | CS | GN | 32 | ≤0.25 | 1 | 1 |
|  | pae41 | CS | GN | 64 | 0.5 | 2 | 1 |
|  | pae42 | CR | GN | 64 | >8 | >8 | 4 |
| *P. mirabilis (n=20)* | pmi1 | NA | GN | 8 | ≤0.25 | 8 | 1 |
|  | pmi2 | NA | GN | 16 | ≤0.25 | 1 | >8 |
|  | pmi3 | NA | GN | 8 | ≤0.25 | 2 | >8 |
|  | pmi4 | NA | GN | 8 | ≤0.25 | 1 | >8 |
|  | pmi5 | NA | GN | 16 | ≤0.25 | 0.5 | >8 |
|  | pmi6 | NA | GN | 8 | ≤0.25 | 1 | 1 |
|  | pmi7 | NA | GN | 4 | 2 | 0.5 | >8 |
|  | pmi8 | NA | GN | 16 | ≤0.25 | ≤0.25 | >8 |
|  | pmi9 | NA | GN | 16 | ≤0.25 | 1 | ≤0.12 |
|  | pmi10 | NA | GN | 16 | ≤0.25 | 4 | 4 |
|  | pmi11 | NA | GN | 8 | ≤0.25 | 1 | 0.5 |
|  | pmi12 | NA | GN | 8 | ≤0.25 | 1 | 0.5 |
|  | pmi13 | NA | GN | 16 | ≤0.25 | 2 | >8 |
|  | pmi14 | NA | GN | 8 | ≤0.25 | 0.5 | 8 |
|  | pmi15 | NA | GN | 2 | ≤0.25 | 0.5 | 1 |
|  | pmi16 | NA | GN | 16 | ≤0.25 | 0.5 | 4 |
|  | pmi17 | NA | GN | 8 | ≤0.25 | 1 | >8 |
|  | pmi18 | NA | GN | 4 | ≤0.25 | 1 | >8 |
|  | pmi19 | NA | GN | 8 | ≤0.25 | 0.5 | >8 |
|  | pmi20 | NA | GN | 16 | ≤0.25 | 0.5 | >8 |
| *E. cloacae (n=20)* | ecl1 | NA | GN | 8 | ≤0.25 | 0.5 | 4 |
|  | ecl2 | NA | GN | 16 | 1 | 2 | 4 |
|  | ecl3 | NA | GN | 8 | ≤0.25 | ≤0.25 | ≤0.12 |
|  | ecl4 | NA | GN | 32 | ≤0.25 | ≤0.25 | >8 |
|  | ecl5 | NA | GN | 16 | ≤0.25 | ≤0.25 | ≤0.12 |
|  | ecl6 | NA | GN | 8 | 1 | 1 | >8 |
|  | ecl7 | NA | GN | 8 | ≤0.25 | 0.5 | 4 |
|  | ecl8 | NA | GN | 8 | ≤0.25 | ≤0.25 | 1 |
|  | ecl9 | NA | GN | 16 | >16 | 8 | 2 |
|  | ecl10 | NA | GN | 16 | >16 | 8 | >8 |
|  | ecl11 | NA | GN | 16 | ≤0.25 | ≤0.25 | ≤0.12 |
|  | ecl12 | NA | GN | 8 | ≤0.25 | 0.5 | ≤0.12 |
|  | ecl13 | NA | GN | 16 | ≤0.25 | ≤0.25 | 0.5 |
|  | ecl14 | NA | GN | 16 | ≤0.25 | ≤0.25 | >8 |
|  | ecl15 | NA | GN | 8 | ≤0.25 | 0.5 | ≤0.12 |
|  | ecl16 | NA | GN | 8 | 2 | 2 | >8 |
|  | ecl17 | NA | GN | 8 | ≤0.25 | ≤0.25 | ≤0.12 |
|  | ecl18 | NA | GN | 8 | 4 | 4 | >8 |
|  | ecl19 | NA | GN | 16 | ≤0.25 | ≤0.25 | ≤0.12 |
|  | ecl20 | NA | GN | 8 | ≤0.25 | ≤0.25 | ≤0.12 |
| *S. epidermidis (n=10)* | sep1 | NA | GP | 2 | NA | NA | 4 |
|  | sep2 | NA | GP | 1 | NA | NA | <=0.12 |
|  | sep3 | NA | GP | ≤00.5 | NA | NA | >4 |
|  | sep4 | NA | GP | 2 | NA | NA | 4 |
|  | sep5 | NA | GP | ≤00.5 | NA | NA | >4 |
|  | sep6 | NA | GP | 2 | NA | NA | 4 |
|  | sep7 | NA | GP | ≤00.5 | NA | NA | 4 |
|  | sep8 | NA | GP | 1 | NA | NA | <=0.12 |
|  | sep9 | NA | GP | ≤00.5 | NA | NA | 4 |
|  | sep10 | NA | GP | 1 | NA | NA | 4 |
| *S. aureus (n=13)* | sau1 | NA | GP | 4 | NA | NA | <=0.12 |
|  | sau2 | NA | GP | 4 | NA | NA | <=0.12 |
|  | sau3 | NA | GP | 4 | NA | NA | 0.25 |
|  | sau4 | NA | GP | 4 | NA | NA | 0.25 |
|  | sau5 | NA | GP | 4 | NA | NA | <=0.12 |
|  | sau6 | NA | GP | 2 | NA | NA | <=0.12 |
|  | sau7 | NA | GP | 4 | NA | NA | <=0.12 |
|  | sau8 | NA | GP | 2 | NA | NA | <=0.12 |
|  | sau9 | NA | GP | 2 | NA | NA | <=0.12 |
|  | sau10 | NA | GP | 2 | NA | NA | 0.25 |
|  | sau11 | NA | GP | 2 | NA | NA | >4 |
|  | sau12 | NA | GP | 2 | NA | NA | >4 |
|  | sau13 | NA | GP | 2 | NA | NA | <=0.12 |
| *E. faecium (n=20)* | efm1 | NA | GP | 8 | NA | NA | >4 |
|  | efm2 | NA | GP | 8 | NA | NA | >4 |
|  | efm3 | NA | GP | 4 | NA | NA | >4 |
|  | efm4 | NA | GP | 4 | NA | NA | >4 |
|  | efm5 | NA | GP | 8 | NA | NA | >4 |
|  | efm6 | NA | GP | 4 | NA | NA | >4 |
|  | efm7 | NA | GP | 4 | NA | NA | >4 |
|  | efm8 | NA | GP | 8 | NA | NA | >4 |
|  | efm9 | NA | GP | 8 | NA | NA | >4 |
|  | efm10 | NA | GP | 16 | NA | NA | >4 |
|  | efm11 | NA | GP | 16 | NA | NA | >4 |
|  | efm12 | NA | GP | 16 | NA | NA | >4 |
|  | efm13 | NA | GP | 8 | NA | NA | >4 |
|  | efm14 | NA | GP | 8 | NA | NA | >4 |
|  | efm15 | NA | GP | 8 | NA | NA | >4 |
|  | efm16 | NA | GP | 8 | NA | NA | >4 |
|  | efm17 | NA | GP | 16 | NA | NA | >4 |
|  | efm18 | NA | GP | 8 | NA | NA | >4 |
|  | efm19 | NA | GP | 8 | NA | NA | >4 |
|  | efm20 | NA | GP | 8 | NA | NA | >4 |
| *E. faecalis (n=10)* | efa1 | NA | GP | 8 | NA | NA | 0.5 |
|  | efa2 | NA | GP | 8 | NA | NA | >4 |
|  | efa3 | NA | GP | 8 | NA | NA | 1 |
|  | efa4 | NA | GP | 16 | NA | NA | 1 |
|  | efa5 | NA | GP | 8 | NA | NA | >4 |
|  | efa6 | NA | GP | 8 | NA | NA | >4 |
|  | efa7 | NA | GP | 8 | NA | NA | 1 |
|  | efa8 | NA | GP | 4 | NA | NA | 0.5 |
|  | efa9 | NA | GP | 8 | NA | NA | 0.5 |
|  | efa10 | NA | GP | 8 | NA | NA | 1 |

^b^ CR: carbapenem-resistant; CS: carbapenem- susceptible; GN: Gram-negative; GP: Gram-positive
